# Editorial: from Brambell to beyond: advancing pig welfare through the five freedoms framework

**DOI:** 10.1186/s40813-025-00449-2

**Published:** 2025-06-24

**Authors:** Mari Heinonen, Elisabeth grosse Beilage

**Affiliations:** 1 University of Helsinki, Helsinki, Finland; 2 University for Veterinary Medicine Hannover, Hannover, Germany

As we commemorate the 60th anniversary of the "Report of the Technical Committee to inquire into the Welfare of Animals kept under intensive livestock husbandry systems" [3], we are presented with an opportunity to reflect on the progress made in pig welfare and to address the challenges that remain. The conceptual framework of the "Five Freedoms" that emerged from this landmark report has fundamentally shaped our understanding of animal welfare and continues to serve as the cornerstone for welfare assessment and improvement strategies globally. This collection of Porcine Health Management invites researchers to contribute manuscripts that advance our understanding of how to fulfil these freedoms in contemporary as well as future swine production systems.

## The five freedoms in modern pig production

The Five Freedoms provide a structured approach to evaluating and improving pig welfare across all production stages. Pig husbandry has changed considerably since the Brambell report, bringing up new challenges in pig welfare. However, we can still place the new challenges under the five freedoms.

Freedom from hunger and thirst necessitates not merely the provision of adequate nutrition but nutrition tailored to specific physiological needs and providing proper feeding and drinking systems facilitating access to feed and water without problems. A deficiency or imbalance in the supply of energy, macronutrients and micronutrients in the diet of pigs is a hazard for many different welfare consequences. The diet composition can influence growth and integrity of all body tissues, metabolic processes as well as the function of the immune system and processes in the brain [5]. While conventional feeding systems often fail the individual nutritional of pigs, precision feeding techniques may improve animal welfare as well as the environmental footprint of pig production [10, 11].

Freedom from discomfort requires holding systems that accommodate the physical and thermal comfort needs of pigs facilitating also a comfortable resting area. The transition from individual stalls to group housing for gestating sows represents a significant advancement [4], yet the design of farrowing accommodations continues to present a complex welfare challenge. Innovative pen designs that balance the sow's need for performing nest building behaviour and movement with piglet protection are emerging, but their wider implementation remains limited in many countries [1].

Freedom from pain, injury, and disease demands preventive health management and prompt intervention when problems arise. Despite considerable progress in disease prevention through improved biosecurity protocols and vaccination strategies, pain management in routine procedures such as tail docking and castration [6] as well as pain therapy in spontaneously occurring diseases [7, 8] remains a prevalent problem in many production systems. The development and validation of practical, pain-mitigating protocols remains an urgent research priority.

Freedom to express normal behaviour requires both space and environmental complexity. The provision of appropriate enrichment materials that facilitate explorative behaviours has been demonstrated to reduce damaging behaviours such as tail biting [12].

Freedom from fear and distress necessitates both appropriate handling practices and social environments that minimize psychological stress. Research by Lucas et al. [9] demonstrated that positive human-animal interactions during early development can significantly reduce fear responses in pigs throughout their lives, underscoring the importance of stockpersonship in welfare outcomes.

## Balancing competing priorities

The implementation of welfare improvements invariably involves navigating complex trade-offs between seemingly competing priorities. This collection aims to explore evidence-based approaches to resolving these apparent conflicts while prioritizing the intrinsic needs of pigs.

The tension between welfare and biosecurity concerns is particularly evident in discussions surrounding environmental enrichment and outdoor access. While straw bedding and other organic materials effectively support natural behaviours, they may also pose biosecurity challenges [2]. Similarly, the conflict between welfare and sustainability goals requires careful consideration. Intensive production systems have historically been optimized for efficiency metrics such as reproductive output and growth rates, sometimes at the expense of welfare indicators. However, emerging research suggests that welfare improvements can contribute positively to sustainability outcomes [11] through reduced mortality, improved feed conversion, and decreased antimicrobial use. This represents a promising area for further investigation. The apparent dichotomy between sow and piglet welfare in farrowing systems exemplifies another critical challenge. Traditional farrowing crates severely restrict sow movement to protect piglets from crushing, creating an ethical dilemma that has proven difficult to resolve. Promising work reviewed by Baxter et al. [1] suggests that with appropriate design and management, both sow freedom of movement and piglet survival can be substantially improved. Finally, the relationship between pig welfare and farmer wellbeing deserves greater attention. Systems that improve animal welfare while increasing labour requirements or compromising worker safety are unlikely to achieve widespread adoption. Research integrating ergonomic principles, labour efficiency, and automation technologies with welfare improvements could help address this challenge.

This collection invites original research, review articles, and case studies that advance our understanding of how to fulfil the Five Freedoms in modern pig production systems.

As we reflect on six decades of progress since the Brambell Report, it is clear that significant advances have been made in our understanding of pig welfare. However, substantial challenges remain in translating this knowledge into widespread practice. By prioritizing the intrinsic needs of pigs while acknowledging practical constraints, this collection aims to contribute to the continued evolution of welfare-centered swine production systems.
